# A balanced transcription between telomerase and the telomeric DNA-binding proteins TRF1, TRF2 and Pot1 in resting, activated, HTLV-1-transformed and Tax-expressing human T lymphocytes

**DOI:** 10.1186/1742-4690-2-77

**Published:** 2005-12-15

**Authors:** Emmanuelle Escoffier, Amélie Rezza, Aude Roborel de Climens, Aurélie Belleville, Louis Gazzolo, Eric Gilson, Madeleine Duc Dodon

**Affiliations:** 1 Virologie Humaine INSERM-U412, Ecole Normale Supérieure de Lyon, IFR 128 BioSciences Lyon-Gerland, 46 Allée d'Italie 69364 Lyon Cedex 07, France; 2 Laboratoire de Biologie Moléculaire de la Cellule, CNRS UMR 5161 Ecole Normale Supérieure de Lyon, IFR 128 BioSciences Lyon-Gerland, 46, allée d'Italie 69364 Lyon Cedex 07, France

## Abstract

**Background:**

The functional state of human telomeres is controlled by telomerase and by a protein complex named shelterin, including the telomeric DNA-binding proteins TRF1, TRF2 and Pot1 involved in telomere capping functions. The expression of *hTERT*, encoding the catalytic subunit of telomerase, plays a crucial role in the control of lymphocyte proliferation by maintaining telomere homeostasis. It has been previously found that *hTERT *activity is down-regulated by the human T cell leukaemia virus type 1 (HTLV-1) Tax protein in HTLV-1 transformed T lymphocytes. In this study, we have examined the effects of Tax expression on the transcriptional profile of telomerase and of shelterin in human T lymphocytes.

**Results:**

We first provide evidence that the up-regulation of *hTERT *transcription in activated CD4+ T lymphocytes is associated with a down-regulation of that of *TERF1, TERF2 *and *POT1 *genes. Next, the down-regulation of *hTERT *transcription by Tax in HTLV-1 transformed or in Tax-expressing T lymphocytes is found to correlate with a significant increase of TRF2 and/or Pot1 mRNAs. Finally, ectopic expression of *hTERT *in one HTLV-1 T cell line induces a marked decrease in the transcription of the *POT1 *gene. Collectively, these observations predict that the increased transcriptional expression of shelterin genes is minimizing the impact on telomere instability induced by the down-regulation of hTERT by Tax.

**Conclusion:**

These findings support the notion that Tax, telomerase and shelterin play a critical role in the proliferation of HTLV-1 transformed T lymphocytes.

## Background

Human telomeres are specialized chromosomal structures that consist of repetitive sequences and a protein complex named shelterin that caps the ends of linear chromosomes [1-3]. Telomeric DNA is mostly composed of double-stranded 5' TTAGGG-3' repeats and terminates with an overhang of single-stranded 3' DNA. In human cells, telomere length is maintained by telomerase (hTERT), a human reverse transcriptase that adds TTAGGG repeats onto the 3' ends of telomeres [4]. hTERT is normally expressed in stem cells and in germ cells, but is present at much reduced levels in many adult somatic cells. As a consequence, loss of telomeric DNA results in replicative senescence through chromosome damage and decrease in cell viability [5]. The shelterin complex is formed by six telomere-specific proteins that provide capping functions and that regulate telomere length [3]. The TRF1, TRF2 and Pot1 subunits bind to telomeric DNA and to the other subunits of the complex, namely the TIN2, TPP1 and Rap1 proteins

Telomerase activity is negatively regulated *in vivo*, at the level of telomere itself, by several shelterin subunits, including TRF1, TIN2, TPP1, Pot1 and Rap1. For instance, Pot1, a single-stranded telomeric DNA-binding protein, behaves as a terminal transducer of the cis-inhibitory effect of the TTAGGG-repeat-binding protein TRF1 [6]. The shelterin subunit TRF2 [7,8] is also involved in a negative regulation of telomere lengthening but by cis-activating rapid deletion events within the telomeric tract [9-11]. Although TRF1 and TRF2 do not directly interact, they are engaged in a dynamic complex for telomere length homeostasis [12].

There is now compelling evidences that telomere modifications seemingly display antagonistic functions in tumorigenesis. On one hand, overexpression of telomerase in cancer cells appears to be crucial for tumor progression thanks to a wealth of studies using mice and cellular models of malignant transformation [13-19]. This is in agreement with the observation that more than 90 % of human tumors overexpress telomerase as compared to the normal matching tissue [20]. On another hand, studies on mice lacking the telomerase RNA gene demonstrate that critical telomere shortening can favor initial stages of cancer formation and cooperates with p53 deficiency to favor carcinogenesis with age [21-23]. In human cells, a burst of telomere instability could also favor tumor formation [21,24-27].

Human T-cell leukemia virus type 1 (HTLV-1) is the etiological agent of adult T-cell leukemia (ATL), which develops after a prolonged period of latency of several decades during which HTLV-1 infected cells proliferate favoring in accumulation of genetic defects and deregulated cell growth [28,29]. Leukemic CD4+ T cells isolated from patients with ATL have been shown to harbor an elevated telomerase activity [30,31]. Likewise, a positive correlation has been established between telomerase activity and development and progression of leukemia [32,33]. Proviral transcription is silent in ATL cells, indicating that viral expression is not directly involved in telomerase activation of ATL cells. We have recently shown that HTLV-1 *in vitro *infected T cells express a low level of telomerase activity and that this decrease is induced by the viral Tax protein [34]. Tax, a regulatory protein that alters the expression or function of numerous genes involved in the proliferation of T cells, is implicated in the initiation of the leukemogenic process [35-39]. In spite of this low level of telomerase activity, HTLV-1 *in vitro *infected T cells and Tax-expressing primary T lymphocytes still continue to proliferate, suggesting the induction of a compensatory mechanism.

In the present study, we have examined the transcriptional profile of the genes encoding hTERT, TRF1, TRF2 and Pot1 in normal T lymphocytes as well as in HTLV-1- transformed and in Tax-expressing T lymphocytes. We observed that the physiological activation of CD4+ T lymphocytes induces an up-regulation of *hTERT *transcription that is correlated with a down-regulation of shelterin subunits (TRF1, TRF2 and Pot1) transcription. Conversely, the down-regulation of *hTERT *transcription mediated by Tax is associated with an up-regulation of *TERF2 *and/or *POT1 *transcription. Furthermore, the ectopic expression of *hTERT *in HTLV-1 transformed T lymphocytes is sufficient to down-regulate the expression of Pot1. Therefore, these results indicate that in normal as well as in HTLV-1 transformed T lymphocytes and in Tax-expressing lymphocytes, the transcriptional balance between hTERT and the shelterin subunits TRF1, TRF2 and Pot1 are regulating telomere homeostasis and cell proliferation.

## Results

### Transcriptional expression of *hTERT*, *POT1*, *TERF1 *and *TERF2 *genes in resting and *in vitro *activated CD4+ T lymphocytes

During *in vitro *as well as *in vivo *activation of T lymphocytes, telomerase expression and activity are known to be tightly regulated [40-42]. To determine whether *POT1, TERF1 *and *TERF2 *genes were submitted to a similar regulation, we analyzed the transcriptional profile of these shelterin genes together with that of *hTERT *in CD4+ T lymphocytes. These cells were isolated from peripheral blood of healthy individuals and either left unstimulated or activated with anti CD3 plus anti CD28 antibodies conjugated beads for 48 hours. Total mRNAs extracted from either resting or activated cells, were then reverse transcribed and analyzed by qPCR with appropriate primers for the expression of *hTERT, POT1, TERF1 *and *TERF2 *genes. For the sake of clarity, the quantitative PCRs assays performed throughout this study were evaluated by using cDNA from Jurkat cells as a standard. As expected, *hTERT *transcription was very low in resting cells, whereas a significant increase (22.6-fold) was observed in activated cells (Table 1). Of note, the increase in the amounts of hTERT mRNAs reaches 65 fold when the latter were cultivated for 10 days in presence of IL2 (data not shown). We next performed a quantitative analysis of the expression of *POT1, TERF1 *and *TERF2 *genes in both cell types. Resting T lymphocytes expressed detectable levels of the respective mRNAs. By contrast, activation of these cells was found to induce a 2.9 to 3.9-fold decrease in the transcription of these shelterin genes. Thus, *in vitro *physiological activation of CD4+ T cells reveals an inverse regulation in the transcription of telomerase and that of *POT1, TERF1 *and *TERF2 *genes.

**Table 1:** Real-time PCR analysis of *hTERT*, *POT1*, *TERF1 *and *TERF2 *gene expression upon activation in freshly isolated CD4+ T lymphocytes.

	Unstimulated	48 h-Stimulated	Fold Activation
hTERT	0.019*	0.43 ± 0.02	22.6
Pot1	10.16 ± 0.02	3.21 ± 0.05	(-) 3.1
TRF1	2.08 ± 0.08	0.70 ± 0.09	(-) 2.9
TRF2	16.69 ± 8.4	4.24 ± 0.03	(-) 3.9

*Values are corrected for the expression of the human housekeeping gene PBGD in cells of each population. Results are expressed as the amount of indicated mRNA relative to PBGD and compared to that found in Jurkat cells referred as 1. Standard deviations are from two determinations performed in triplicate.

### Transcriptional expression of *hTERT*, *POT1*, *TERF1 *and *TERF2 *genes in HTLV-1 transformed and Tax-expressing T lymphocytes

**Figure 1:**
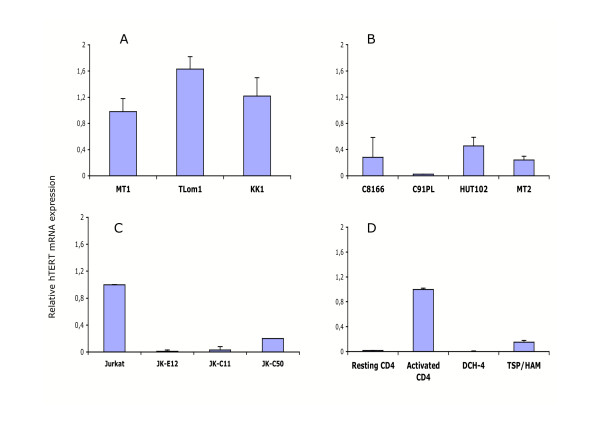
Real time quantitative PCR (qPCR) analysis of hTERT expression in HTLV-1-infected and uninfected T cells. A) ATL cell lines (HTLV-1 infected but negative for Tax expression); B) In vitro transformed cell lines (HTLV-1 infected and positive for Tax expression); C) Jurkat T-cell clones positive for Tax expression (E12, C11 and C50); D) Uninfected primary CD4 T lymphocytes either activated or not (resting); T-cell line (DCH-4) immortalized with a lentivirus vector encoding a Tax-YFP fusion protein; Infected T lymphocytes isolated from patient suffering from TSP/HAM. Cytoplasmic RNAs were isolated, reverse transcribed and cDNA were analyzed by qPCR using primers for hTERT and PBGD. Results are expressed as indicated in legend of table 1. Standard deviations are from at least three determinations performed in duplicate.

It has been previously shown a decrease of telomerase activity (assayed by TRAP assays) in HTLV-1 T cell lines expressing Tax [34]. This decrease was associated with the abitity of Tax to downregulate the hTERT promoter. To further assess the inhibitory effect of Tax on the transcription of the telomerase gene, the levels of hTERT mRNAs in T cell lines, which do not express Tax were compared to those in Tax-expressing T cells. The transcription of *hTERT *was found to be significantly higher in the three cell lines from ATL patients, (in which Tax is undetectable), than in the three *in vitro *HTLV-1 transformed (IL2-independent) T-cells (C91PL, HUT-102, MT2), which are expressing Tax and producing viral particles (Fig. 1, compare A and B). Likewise, *hTERT *transcription decreased in the three Jurkat T-cell clones expressing only Tax, when compared to the parental cells (Fig. 1C). The transcription of the telomerase gene was next analyzed in IL2-dependent CD4+ DCH4 T-cells, obtained after transduction of activated primary human CD4+ cells with a lentivirus vector encoding Tax and in peripheral blood T lymphocytes isolated from a TSP/HAM patient, which are known to express Tax. In both cell types, the amount of hTERT mRNA was ten-fold less than that in normal activated CD4+ T lymphocytes (Fig. 1D). Together, these observations underline a direct correlation, between Tax expression and *hTERT *transcriptional repression in T cells.

**Figure 2:**
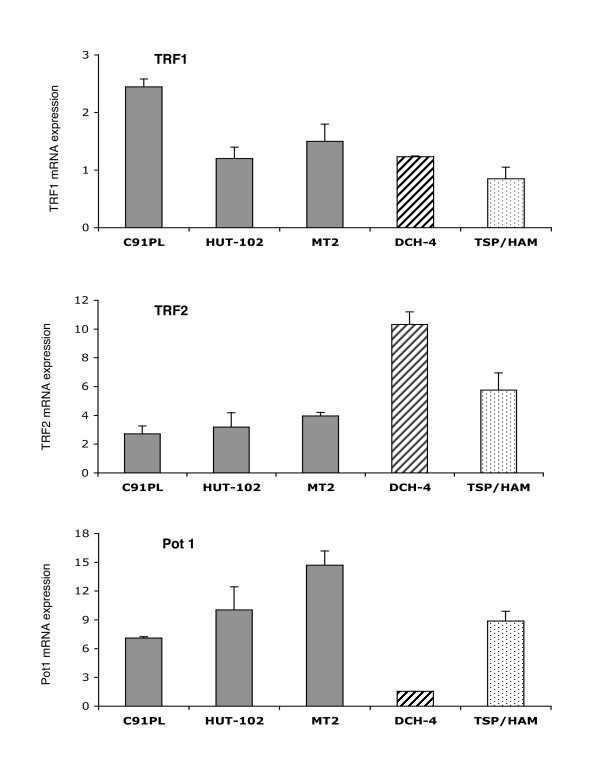
Analysis of *TERF1*, *TERF2 *and *POT1 *gene expression in T-cell lines expressing Tax. Cytoplasmic RNAs were isolated, reverse transcribed and cDNA were analyzed by qPCR using specific primers. Results are expressed as the amount of indicated mRNA relative to PBGD. Each quantification was compared to that obtained with Jurkat cells, referred as 1. Standard deviations are from at least three determinations performed in duplicate.

As indicated above, in resting T lymphocytes, a low level of telomerase gene transcription correlates with a high level of shelterin gene transcription. Therefore, these results prompted us to test whether the inhibition of hTERT transcription by Tax could lead to an enhancement of the transcription of the shelterin genes. To that purpose, we analyzed the transcriptional profile of *TERF1, TERF2 *and *POT1 *in the HTLV-1 transformed T-cell lines (C91PL, HUT-102, MT2). Remarkably, while the transcription of *TERF1 *and of *TERF2 *underwent an increase of 1.2 to 2.4, and of 2.7 to 4.0, respectively, the transcription of *POT1 *rose at a much higher level (Fig. 2). Indeed, the amount of Pot1 in C91PL, HUT-102 and MT2 cells was respectively 7.1-, 10.0- and 14.7-fold. In the DCH4 and in the TSP/HAM cells, only a slight increase of *TERF1 *transcription was observed, but lower than that in C91PL cells (Fig. 2). Whereas the transcription of the *TERF2 *gene was greatly enhanced in these cells (10.3 for the DCH4 cells and 5.8 for the TSP/HAM cells), that of the *POT1 *gene increased 8.9-fold in the TSP/HAM cells, but not in DCH4 cells. Overall these results underline that the down-regulation of *hTERT *transcription in HTLV-1-T cell lines and in Tax-expressing T lymphocytes correlates with an increased transcription of shelterin genes, and more particularly that of *POT1 *and *TERF2 *genes. Collectively, these results validate the balanced transcription between telomerase and the telomeric DNA-binding proteins TRF1, TRF2 and Pot1.

### Effect of ectopic expression of *hTERT *in HTLV-1 transformed T lymphocytes on shelterin gene expression

**Figure 3:**
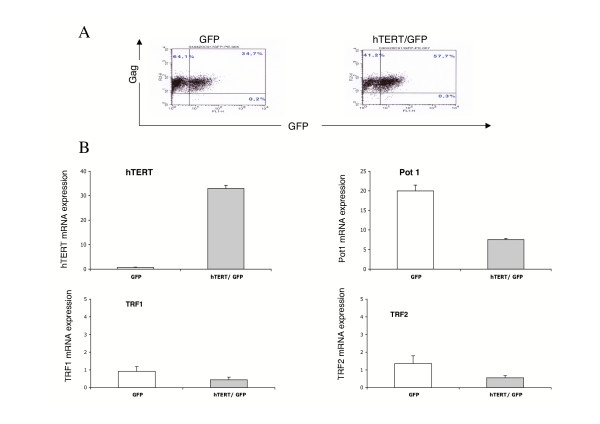
Characterization of C91PL cells over-expressing *hTERT *gene. Cells were transduced with a lentivirus vector encoding GFP with or without hTERT A) Flow cytometry determination of the expression of GFP and p19_gag_. The percentage of cells in each quadrant is indicated. B) Analysis of *hTERT*, and of telomere protein gene expression in hTERT/GFP transduced C91PL cells (grey bars) and in control GFP transduced C91PL (white bars) was performed and quantified as in Figure 2.

The above results showing an inverse correlation of hTERT transcription with that of shelterin genes in T lymphocytes proposes the possibility that *hTERT *negatively regulates the transcription of the shelterin genes in these cells. To test this hypothesis, we studied the effect of an ectopic *hTERT *expression in C91PL cells, in which the low level of hTERT transcription is associated with a high level of *POT1 *transcription. To that purpose, C91PL cells were transduced with either the pWPIR-hTERT-GFP or the control pWPIR-GFP lentiviral vectors. In these vectors, the transcription of the hTERT-IRES-GFP and that of the IRES-GFP sequences were under the control of an heterologous promoter (EF1), which is not known to be down-regulated by Tax. Ten days after transduction, cells were analyzed for GFP expression and for transcription of *hTERT *and shelterin genes. First, FACS analysis showed that a significant percentage (more than 50%) of both types of transduced cells were GFP positive (Fig 3A). Next, quantitative RT-PCR analysis showed that in hTERT-GFP-transduced cells, a 47.6-fold increase of *hTERT *transcription was observed, as compared to GFP control cells (Fig. 3B). No significant modification in the proliferation rate of the cell population over-expressing *hTERT *was observed. Finally, a more than 2-fold decrease in the levels of *TERF1*, *TERF2 *and *POT1 *transcripts were observed. Thus, Pot1 mRNAs, which are the most abundant in GFP-transduced cells, were found to decrease by 2.7-fold in the hTERT-GFP-transduced C91PL cells. These results confirm that the transcriptional expression of shelterin genes appears to be down-regulated by *hTERT*. They further indicate that in HTLV-1 T cell lines Tax expression does not interfere with this regulatory mechanism.

## Discussion

**Figure 4:**
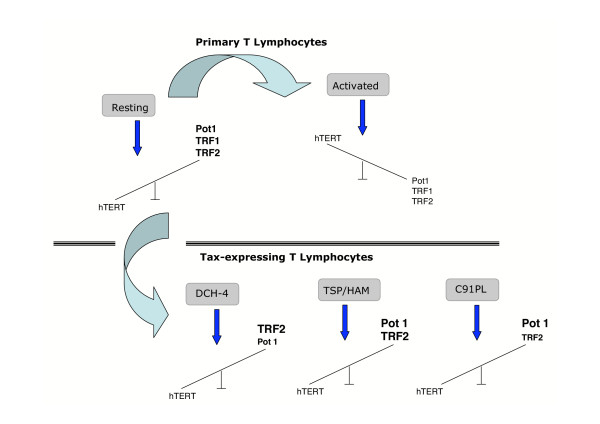
A model for hTERT and shelterin gene expression in T lymphocytes. In the upper panel, CD4+ T lymphocytes were either resting or activated with a cocktail of anti CD3/anti CD28 antibodies. In the lower panel, three types of Tax-expressing T lymphocytes were represented. The model is based on the telomeric transcriptional profile defined as a balance between *hTERT *on one hand, and shelterin (*POT1*, *TERF1 *and *TERF2*) gene expression on the other hand.

In this study, we provide evidence that, during the activation of human CD4+ T lymphocytes, the increased transcription of the gene encoding the telomerase catalytic subunit is accompanied by a decreased transcription of the genes encoding three subunits (TRF1, TRF2 and Pot1) belonging to the shelterin complex. Since the products of these genes are known to inhibit telomere length, telomere homeostasis in activated T lymphocytes appears to be regulated both by telomerase induction and by shelterin repression. We therefore propose that, during T cell activation, the functional state of telomeres is regulated by a change in the balance between the expression of *hTERT *and that of shelterin genes (Figure 4, upper panel). Human CD4+ T lymphocytes are the main targets of HTLV-1 transformation and the viral regulatory Tax has also been shown to inhibit telomerase activity [34]. We indeed show that transcription of the telomerase gene is inhibited in three *in vitro *HTLV-1 transformed T cell lines as well as in Tax-expressing DCH4 T lymphocytes and in TSP/HAM T lymphocytes. We next observe that the Tax-induced decrease of hTERT mRNAs in these cells corresponds to an increase in the overall amount of the *TERF1, TERF2 *and *Pot1 *transcripts. Thus it appears that telomere homeostasis in HTLV-1 T cell lines and in Tax-expressing lymphocytes is regulated both by telomerase repression and by shelterin induction (Fig. 4, lower panel). Collectively, these results validate a balanced transcription between telomerase and the telomeric DNA-binding proteins TRF1, TRF2 and POT1 in normal, activated as well as in HTLV-1 infected and in Tax-expressing T lymphocytes. They therefore plead for a regulatory mechanism controlling this balance. Thus, the observation that ectopic expression of hTERT in HTLV-1 T cells leads to a decrease in the transcription of these genes, suggests that hTERT is implicated in their negative transcription. It would be worth to determine whether the ectopic expression of any shelterin subunit would lead to a down-regulation of hTERT transcription. Interestingly, HTLV-1 transformed T cells and Tax expressing T lymphocytes are sharing with uninfected resting T lymphocytes, the same transcriptional telomeric profile. It therefore appears that the transcriptional balance between hTERT and the three shelterin subunits is involved not only in regulating telomere homeostasis, but also in sustaining HTLV-1-induced cellular proliferation.

As indicated above, while the overall transcription of the shelterin genes were found to increase in HTLV-1 T cell lines and in Tax-expressing T lymphocytes, the transcription of each shelterin subunit was not affected to a similar extent in each cell type. Thus, the transcription of the *POT1 *gene was enhanced to a higher level than that of the *TERF1 *and *TERF2 *genes in the HTLV-1 T cell lines. Likewise, only *TERF2 *was found to be up-transcribed in DCH4 cells, whereas the transcription of both *TERF2 *and *POT1 *was significantly increased in TSP/HAM cells (Fig. 4, lower panel). It is plausible that these differences might be linked to their *in vitro/in vivo *derivation and/or to selection pressures in culture. Whatsoever, these data suggest that Tax is not intervening in the modulation of this balanced transcription. Indeed, the observation that Pot1 transcription decreased in C91PL cells over-expressing hTERT implies that the activity of Tax on the telomeric machinery is restricted to its inhibitory effect on hTERT transcription.

Cancer cells commonly up-regulate telomerase, which is consistent with telomerase conferring a strong selective advantage for continued growth of malignant cells [43]. As a matter of fact, telomerase is highly expressed in patients with the acute type of ATL [33]. In these ATL cells, proviral transcription is silent, underlining that viral genome expression is crucial only at the onset of the leukemogenic process. The present study suggests that in infected T cells in which proviral expression is active, the increased expression of TRF2 and/or Pot1, involved in telomere capping functions, could trigger protective mechanisms that compensate the decrease of telomerase expression. Thus, by playing an important role in telomere homeostasis, the shelterin proteins are allowing the proliferation of HTLV-1 infected T cells or Tax-expressing T lymphocytes. Consequently, we anticipate that a transcriptional decrease of these telomeric proteins coupled with telomerase reactivation which might occur at a time, when Tax is no more expressed, would contribute to the emergence of telomerase-positive acute leukemic cells. Interestingly, recent studies have provided new evidence that telomerase enhances expression of growth-controlling genes to confer additional pivotal functions in tumor progression other than telomere length maintenance [44]. Although more work is needed to elucidate the cellular and molecular mechanisms of this telomere dysfunction during the HTLV-1-induced leukemogenic process, the present observations reveal new links between Tax, telomerase and shelterin, that might play a key role in the maintenance and in the proliferation of HTLV-1 infected T lymphocytes.

## Methods

### Cells

The HTLV-1 T-cell lines (IL-2 independent) C91PL [45], MT2 [46], HUT102 [47] and C8166 [48] have been described elsewhere. The HTLV-1 T-cell lines either (IL-2 dependent) KK1, or (IL-2 independent), MT1 and TLom1 were generously provided by Dr. N. Mori [49]. The DCH4 cells, (kind gift by Dr. D. Derse), were established by transduction of activated, primary human CD4+T cells with a lentivirus vector encoding an HTLV-1 Tax-enhanced yellow fluorescent protein fusion [50]. Three clones of Jurkat T cells stably producing Tax (E12, C11, C50) have been used in this study [51]. T lymphocytes isolated from one TSP/HAM patient (CJ) were kindly provided by Dr. A. Gessain (Paris, France). These lymphocytes and the cell lines KK1 and DCH4 were cultivated in RPMI 1640 (Invitrogen) with 10% heat-inactivated fetal calf serum (FCS) 100 U/ml recombinant human IL-2 (rhIL-2) and supplemented with 100 IU/ml of penicillin and 50 *μ*g/ml of streptomycin. The Jurkat parental as well as the Jurkat Tax-expressing clones, C91PL, MT2, HUT102, MT1 and TLom1 cell lines were maintained in complete RPMI-1640 medium with 10% FCS, without rhIL-2. The human 293T and rhabdomyosarcoma TE cells were cultured in Dulbecco's minimum Eagle medium (DMEM, Invitrogen) supplemented with 10% FCS and antibiotics.

Primary peripheral blood lymphocytes from healthy volunteers were isolated by Ficoll density gradient centrifugation. Then, CD4+ T cell subsets were negatively selected using magnetic beads (Stem Cell Technologies, Vancouver, BC) according to the manufacturer instructions. Purified CD4+ T cells were activated with anti CD3/anti CD28 antibody coated beads (Dynal Biotech, Lake Success, NY) and maintained in RPMI-1640 with 10% FCS and rhIL-2.

### Lentiviral constructs and hTERT transduction

The pWPIR-GFP HIV-derived vector, obtained from Didier Trono, contained the IRES-GFP (enhanced GFP as a marker gene) under the control of the EF1 (human elongation factor 1 alpha) promoter [52]. The pWPIR-hTERT-GFP construct was generated by inserting the hTERT cDNA upstream of the IRES in order to allow individual translation of the bicistronic mRNA containing both hTERT and GFP (hTERT-IRES-GFP). Helper-free recombinant lentiviruses were produced after transfection of 293T cells with the three following constructs (1) a packaging plasmid, pCMVR8.91; (2) a transfer vector, pWPIR-hTERT-GFP or pWPIR-GFP; (3) an envelope expression plasmid, pCMV-VSVG. Twenty to 30 hours later, the supernatant was harvested, filtered through a 0.45-*μ*m membrane and aliquots were stored at -80°C. Titres of virus stocks (from 3 to 5 × 10^5 ^transducing units per ml) were determined by transduction of human rhabdomyosarcoma TE cells with serially diluted viral supernatant and analysis five days later on a FACScan instrument (Becton Dickinson, Mountain View, CA). C91PL cells cultured for 1–2 hours in presence of polybrene (8 μg/ml) were then incubated overnight with virus supernatant at a multiplicity of infection of 2. Analysis of GFP-expressing cells was performed on a FACScan. Data were analyzed with the Cell Quest program ((Becton Dickinson).

### Real-time polymerase chain reaction amplification

Total cellular RNAs were isolated from cells using Qiagen RNeasy purification kits (Qiagen, Alameda, CA) according to the manufacturer's instructions. To reduce the amount of DNA originating from lysis, samples were treated with RNase-free DNase (10 U/*μ*l, Qiagen) for 30 min at 20°C and then for 15 min at 65°C. Five-hundred ng of RNA sample were reverse transcribed by using oligo(dT)12–18 and Superscript II (InVitrogen Life technologies, Frederick, MD). Reverse transcription was performed for 50 min at 42°C. The total cDNA volume of 20 *μ*l was frozen until real-time quantitative PCR was performed. After thawing for PCR experiments, the cDNA was diluted in distilled water and 2 *μ*l of diluted cDNA was used for each PCR reaction. The real-time quantitative PCR (qPCR) was performed in special lightcycler capillaries (Roche) with a lightcycler Instrument (Roche), by using the LightCycler-FastStart reaction Mix SYBR-Green kit (Roche). The following specific primers were used to detect: PBGD, sense 5'-GGAATGCATGTATGCTGTGG-3' and antisense, 5'-CAGGTACAGTTGCCCATCC-3', Tax_HTLV-1_sense, 5'-GTTGTATGAGTGATTGGCGGGGTAA-3' and antisense, 5'-TGTTTGGAGACTGTGTACAAGGCG-3', hTERT sense, 5'-TGTTTCTGGATTTGCAGGTG-3' and antisense, 5'-GTTCTTGGCTTTCAGGATGG-3', Pot1 sense, 5'-TGGGTATTGTACCCCTCCAA-3' and antisense, 5'-GATGAAGCATTCCAACCACGG-3'. TRF1 sense,5'-GCTGTTTGTATGGAAAATGGC-3' and antisense: 5'-CCGCTGCCTTCATTAGAAAG-3', TRF2 sense, 5'-GACCTTCCAGCAGAAGATGC-3' and antisense, 5'-GTTGGAGGATTCCGTAGCTG-3'. The thermal cycling conditions consisted of 40 cycles at 95°C for 10 sec, 61°C for 5 sec, 72°C for 10 sec. The fluorescence signal increase of SYBR-GREEN was automatically detected during the 72°C phase of the PCR. Omission of reverse transcriptase in the RT-PCR protocol led to a failure of target gene amplification in the positive controls. Light cycler PCR data were analyzed using LightCycler Data software (Idaho Technology). The software first normalizes each sample by background subtraction of initial cycles. A fluorescence threshold is then set at 5% full scale, and the software determines the cycle number at which each sample reached this threshold. The fluorescence threshold cycle number correlates inversely with the log of initial template concentration. A standard calibration curve was performed by using cDNA from Jurkat cells. The levels of PBGD transcripts were used to normalize the amount of cDNA in each sample.

## Competing interests

The author(s) declare that they have no competing interests.
